# Supplementary material for: People overlook subtractive solutions to mental health problems
Source: Commun Psychol. 2025 Aug 20;3:128. doi: 10.1038/s44271-025-00312-8 (PMC12368146; doi:10.1038/s44271-025-00312-8)
Supplement: Supplementary file 2 — Supplemental results [file 44271_2025_312_MOESM2_ESM.pdf]

Results

Study 1

We ran a linear mixed model predicting number of solutions with fixed effects for solution type (additive vs. subtractive), the number of activities the person in the vignette was already engaged in (high vs. low), vignette gender, participant gender and participant age. Participant ID was entered as a random effect.

There was a main effect of Solution, $F(1, 1652) = 63.061$, $p < .001$, $partR^2 = .005$, 95% CI [0.013, 0.001]. Participants offered more additive ($EMMean = 2.801$; $SE = 0.060$) than subtractive solutions ($EMMean = 0.931$; $SE = .060$).

There was also a significant interaction between Solution and Participant Gender, $F(1, 1652) = 6.694$, $p = .010$, $partR^2 = .001$, 95% CI [0.005, 0.000]. However, both men, $b = 1.69$, $SE = .079$, $t(1652) = 22.945$, $p < .001$, and women, $b = 2.05$, $SE = .079$, $t(1652) = 26.716$, $p < .001$, suggested more additive than subtractive solutions.

There was also a significant interaction between Solution and Participant Age, $F(1, 1652) = 5.647$, $p = .018$, $partR^2 = .000$, 95% CI [0.003, 0.000]. Simple slopes analysis suggested that the number of additive solutions suggested increased with age, $b = 0.016$, $SE = .005$, $t(367.78) = 3.280$, $p = .001$, whereas there was no association between age and subtractive solutions, $b = 0.003$, $SE = .005$, $t(367.78) = 0.6718$, $p = .502$.

There was also a significant interaction between Solution and Number of Activities, $F(1, 1652) = 3.989$, $p = .046$, $partR^2 = .000$, 95% CI [0.003, 0.000]. However, significantly more additive solutions were recommended at high, $b = 2.15$, $SE = .075$, $t(1652) = 28.550$, $p = <$ .001, and low, $b = 1.59$, $SE = .075$, $t(1652) = 21.180$, $p < .001$, levels of existing activity.

Study 2

We ran two models, the first included fixed effects for solution type, participant gender and vignette gender. The second model included fixed effects for solution type, participant age and vignette age.

Gender

There was a main effect of Solution, $F(1, 1419.12) = 1291.66$, $p < .001$, $partR^2 = .073$, 95% CI [0.088, 0.051]. Participants offered more additive (*EMMean* = 1.516; *SE* = .029) than subtractive solutions (*EMMean* = 0.447; *SE* = .029).

There was also an interaction between Solution and Participant Gender, $F(1, 1419.12) = 22.592$, $p < .001$, $partR^2 = .006$, 95% CI [0.016, 0.001], however, both men, $b = 0.927$, $SE = .043$, $t(1420) = 21.846$, $p < .001$, and women, $b = 1.210$, $SE = .042$, $t(1421) = 29.051$, $p < .001$, suggested more additive than subtractive solutions.

The three way interaction between Participant and Vignette Gender and Solution was not significant, $F(1, 1418.90) = 2.047$, $p = .153$, $partR^2 = .001$, 95% CI [0.006, 0.000].

Age

Besides the main effect of Solution, $F(1, 1418.95) = 65.889$, $p < .001$, $partR^2 = .019$, 95% CI [0.034, 0.008], there was also a significant interaction between Solution and Participant Age, $F(1, 1418.90) = 4.7202$, $p = .030$, $partR^2 = .003$, 95% CI [0.011, 0.000]. Participants became more additive the older they were, $b = 0.006$, $SE = .003$, $t(365.58) = 2.304$, $p = .022$. There was no association between age and number of subtractive solutions recommended, $b = 0.000$, $SE = .003$, $t(365.22) = 0.107$, $p = .915$.

There was no significant interaction between Solution with Vignette Age, $F(1, 1418.95) = 0.380$, $p = .538$, $partR^2 = .000$, 95% CI [0.004, 0.000], or a three way interaction between

Participant and Vignette Age and Solution, $F(1, 1419.12) = 0.064$, $p = .801$, $partR^2 = .000$, 95% CI [0.003, 0.000].

Study 3

We ran an LLM that included fixed effects for Solution and Number of Activities already engaged in, as well as the interaction between these effects.

There was a main effect of Solution, $F(1, 119) = 80.837$, $p < .001$, $partR^2 = .229$, 95% CI [0.320, 0.146]. Users on reddit offered more additive (*EMMean* = 1.860; *SE* = .113) than subtractive solutions (*EMMean* = 0.174; *SE* = .113). There was no main effect of Activities, $F(1, 119) = 0.000$, $p = .993$, $partR^2 = .000$, 95% CI [0.022, 0.000], or interaction with Solution, $F(1, 119) = 0.103$, $p = ..749$, $partR^2 = .000$, 95% CI [0.022, 0.000].

Study 4

We ran three LLMs that each included fixed effects for Condition (whether the vignette presented only additive solutions, subtractive solutions or an equal mixture of the two), vignette gender and participant gender. It also included participant ID and vignette number (1-3) as random effects. These models predicted either participant ratings of solution effectiveness, acceptability, or the extent to which participants would adopt them themselves.

Effectiveness

The model predicting effectiveness had a main effect of Condition, $F(2, 2840) = 190.429$, $p < .001$, $partR^2$ for the effect comparing subtractive to additive conditions = .009, 95% CI [0.017, 0.004], $partR^2$ for the effect comparing mixed to additive conditions = .002, 95% CI [0.007, 0.000]. As indicated by the $partR^2$ effects, additive solutions were rated as more effective than mixed, $b = 0.338$, $SE = 0.038$, $t(2840) = 9.003$, $p < .001$, and subtractive solutions, $b = 0.732$, $SE = .038$, $t(2840) = 19.497$, $p < .001$. Mixed solutions were also rated

as more effective than subtractive solutions, $b = 0.394$, $SE = 0.038$, $t(2840) = 10.494$, $p <$ .001.

There was also a significant Condition by Participant Gender interaction, $F(2, 2840) = 6.553$, $p = .001$, $partR^2$ for the interaction between Gender and the contrast between subtractive vs additive conditions = .000, 95% CI [0.003, 0.000], $partR^2$ for the interaction between Gender and contrast between mixed vs. additive conditions = .000, 95% CI [0.002, 0.000]. However, both men and women reported that additive solutions were more effective than mixed and subtractive solutions and that mixed solutions were more effective than subtractive solutions (all p's < .001)

Acceptability

The model predicting acceptability had a main effect of Condition, $F(2, 2839.9) = 120.026$, $p < .001$, $partR^2$ for the effect comparing subtractive to additive conditions = .011, 95% CI [0.018, 0.005], $partR^2$ for the effect comparing mixed to additive conditions = .003, 95% CI [0.007, 0.000]. As indicated by the $partR^2$ effects, additive solutions were rated as more acceptable than mixed, $b = 0.259$, $SE = 0.038$, $t(2840) = 6.727$, $p < .001$, and subtractive solutions, $b = 0.594$, $SE = .038$, $t(2840) = 15.451$, $p < .001$. Mixed solutions were also rated as more acceptable than subtractive solutions, $b = 0.335$, $SE = 0.038$, $t(2840) = 8.724$, $p <$ .001.

There was also a significant Condition by Participant Gender interaction, $F(2, 2840) = 9.413$, $p < .001$, $partR^2$ for the interaction between Gender and the contrast between subtractive vs additive conditions = .002, 95% CI [0.005, 0.000], $partR^2$ for the interaction between Gender and contrast between mixed vs. additive conditions = .000, 95% CI [0.002, 0.000]. However, both men and women reported that additive solutions were more acceptable than mixed and

subtractive solutions and that mixed solutions were more acceptable than subtractive solutions (all p's < .001)

Relatability

The model predicting the extent to which participants would adopt the solutions themselves had a main effect of Condition, $F(2, 2839.9) = 131.907$, $p < .001$, $partR^2$ for the effect comparing subtractive to additive conditions = .013, 95% CI [0.021, 0.006], $partR^2$ for the effect comparing mixed to additive conditions = .004, 95% CI [0.009, 0.001]. As indicated by the $partR^2$ effects, additive solutions were rated as more relevant than mixed, $b = 0.376$, $SE = 0.049$, $t(2840) = 7.642$, $p < .001$, and subtractive solutions, $b = 0.800$, $SE = .049$, $t(2840) = 16.233$, $p < .001$. Participants also rated mixed solutions as more relevant to themselves than subtractive solutions, $b = 0.423$, $SE = 0.038$, $t(2840) = 8.724$, $p < .001$.

There was also a significant Condition by Participant Gender interaction, $F(2, 2840) = 8.357$, $p < .001$, $partR^2$ for the interaction between Gender and the contrast between subtractive vs additive conditions = .002, 95% CI [0.006, 0.000], $partR^2$ for the interaction between Gender and contrast between mixed vs. additive conditions = .000, 95% CI [0.002, 0.000]. However, both men and women reported that they more likely to use additive solutions than subtractive solutions and mixed solutions more than subtractive solutions (all p's < .001)

Study 5

We ran two linear mixed models with fixed effects for solution type (additive vs. subtractive), vignette gender, participant gender and participant age. Participant ID was entered as a random effect. The first model predicted the number of solutions that were recommended. The second model predicted the number of solutions participants ranked within their top 5 most effective solutions.

Number of solutions recommended

There was a main effect of Solution, $F(1, 472.69) = 174.722, p < .001, partR^2 = .009$, 95% CI [0.029, 0.000]. Participants offered more additive (*EMMean* = 3.290; *SE* = .085) than subtractive solutions (*EMMean* = 2.690; *SE* = .085). There was also a significant Solution by Participant Gender interaction, $F(1, 472.69) = 6.844, p = .009, partR^2 = .001$, 95% CI [0.013, 0.000]. However, both Men, $b = 0.481$, $SE = .064$, $t(472) = 7.497, p < .001$, and Women, $b = 0.719$, $SE = .064$, $t(472) = 11.197, p < .001$, suggested significantly more additive than subtractive solutions and did not differ significantly from one another in how additive or subtractive they were (smallest $p = .725$, for difference in number of subtractive solutions suggested).

Ranking of solutions

There was a main effect of Solution, $F(1, 447.42) = 75.414, p < .001, partR^2 = .014$, 95% CI [0.039, 0.002]. Participants included more additive solutions in their top 5 most effective solutions (*EMMean* = 2.44; *SE* = .042) than subtractive solutions (*EMMean* = 1.93; *SE* = .042). There was also a significant Solution by Participant Gender interaction, $F(1, 447.42) = 4.8863, p = .028, partR^2 = .005$, 95% CI [0.022, 0.000]. However, both Men, $b = 0.379$, $SE = .083$, $t(447) = 4.589, p < .001$, and Women, $b = 0.638$, $SE = .083$, $t(447) = 7.687, p < .001$, placed more additive than subtractive solutions in their top 5 most effective solutions and did not differ significantly from one another in how additive or subtractive they were (smallest $p = .347$, for difference in number of additive solutions suggested).

Study 6

We repeated the models used in Study 5.

Number of solutions recommended

There was a main effect of Solution, $F(1, 517.07) = 235.018, p < .001, partR^2 = .051$, 95% CI [0.087, 0.024]. Participants offered more additive (*EMMean* = 3.57; *SE* = .086) than

subtractive solutions (*EMMean* = 2.91; *SE* = .086). There was also a significant Solution by Participant Gender interaction, $F(1, 517.07) = 12.014$, $p < .001$, $partR^2 = .008$, 95% CI [0.025, 0.000]. However, both Men, $b = 0.513$, $SE = .063$, $t(517) = 8.204$, $p < .001$, and Women, $b = 0.812$, $SE = .060$, $t(517) = 13.606$, $p < .001$, suggested significantly more additive than subtractive solutions and did not differ significantly from one another in how additive or subtractive they were (smallest $p = .166$, for difference in number of subtractive solutions suggested).

Ranking of solutions

There was a main effect of Solution, $F(1, 667) = 22.652$, $p < .001$, $partR^2 = .103$, 95% CI [0.149, 0.065]. Participants included more additive solutions in their top 5 most effective solutions (*EMMean* = 2.45; *SE* = .042) than subtractive solutions (*EMMean* = 2.17; *SE* = .042). There was also a significant Solution by Participant Gender interaction, $F(1, 667) = 44.191$, $p < .001$, $partR^2 = .042$, 95% CI [0.076, 0.018]. Women put more additive solutions in the top 5 most effective solutions than they did subtractive solutions, $b = 0.680$, $SE = .082$, $t(500) = 8.264$, $p < .001$, whereas Men did not differ in the number of additive and subtractive solutions they placed in the top 5 ($b = -0.113$, $SE = .086$, $t(500) = -1.305$, $p =.560$.

Study 6.5

In this reanalysis of Study 5 and 6 data, two models were tested. Both included random effects for participant ID and counterbalance condition (or study). The first model predicted number of solutions recommended and included fixed effects for solution type, participant gender and harm type (positive vs. negative harms). The second model predicted number of solutions included in the top 5 most effective and included the same fixed effects as the first model.

Number of solutions recommended

There was a main effect of Solution, $F(1, 998.73) = 395.17$, $p < .001$, $partR^2 = .058$, 95% CI [0.084, 0.036]. Participants offered more additive (*EMMean* = 3.49; *SE* = .123) than subtractive solutions (*EMMean* = 2.77; *SE* = .123).

There was also an interaction between Solution and Participant Gender, $F(1, 998.73) = 16.212$, $p < .001$, $partR^2 = .002$, 95% CI [0.009, 0.000]. However, both men, $b = 0.578$, $SE = .052$, $t(998) = 11.086$, $p < .001$, and women, $b = 0.871$, $SE = .052$, $t(998) = 17.096$, $p < .001$, offered more additive than subtractive solutions.

There was also a significant Solution by Harm interaction, $F(1, 998.73) = 86.156$, $p < .001$, $partR^2 = .022$, 95% CI [.022, .002]. Participants suggested more additive than subtractive solutions to people who were engaged in more negative harms – activities that harm their mental health through the absence of something beneficial (e.g., not exercising) – than positive harms – activities that harm them through the presence of something detrimental (e.g., smoking), $b = 1.063$, $SE = .052$, $t(998) = 20.605$, $p < .001$. The same was also true for people who engaged in more positive than negative harms, $b = 0.386$, $SE = .052$, $t(998) = 7.498$, $p < .001$. However, compared to people engaged in more positive harms, people engaged in more negative harms were recommended more additive solutions, $b = 0.258$, $SE = .052$, $t(999) = 5.003$, $p < .001$, and fewer subtractive solutions, $b = -0.418$, $SE = .052$, $t(999) = -8.107$, $p < .001$.

Ranking of solutions

There was a main effect of Solution, $F(1, 1278) = 116.655$, $p < .001$, $partR^2 = .105$, 95% CI [0.137, 0.076]. Participants ranked more additive (*EMMean* = 2.5; *SE* = .069) than subtractive solutions (*EMMean* = 2.2; *SE* = .069) in their top 5 most effective.

There was also an interaction between Solution and Participant Gender, $F(1, 1278) = 39.767$, $p < .001$, $partR^2 = .017$, 95% CI [0.034, 0.006]. However, both men, $b = 0.209$, $SE = .066$,

$t(958) = 3.139$, $p = .002$, and women, $b = 0.794$, $SE = .066$, $t(958) = 12.249$, $p < .001$, ranked more additive than negative solutions in their top 5 most effective solutions.

There was also a significant Solution by Harm interaction, $F(1, 1278) = 46.205$, $p < .001$, $partR^2 = .020$, 95% CI [.038, .008]. Participants ranked more additive than subtractive solutions in their top 5 for people who were engaged in more negative harms than positive harm, $b = 0.817$, $SE = .066$, $t(958) = 12.444$, $p < .001$. The same was also true for people who engaged in more positive than negative harms, $b = 0.186$, $SE = .066$, $t(958) = 2.831$, $p = .005$. Again, compared to people engaged in more positive harms, for people engaged in more negative harms additive solutions were seen as more effective, $b = 0.294$, $SE = .066$, $t(963) = 4.475$, $p < .001$, and subtractive solutions were seen as less effective, $b = -0.337$, $SE = .066$, $t(963) = -5.137$, $p < .001$.

Study 7

Four models were tested. The first two models included fixed effects for solution type, participant gender and advice recipient (oneself, a friend or a stranger). As before, one model predicted number of solutions given and another predicted number of solutions included within the rankings of top 5 most effective.

In a second set of models, we selected only the data from when advice was given to a stranger. These models included fixed effects for solution type, participant gender and vignette gender and also harm type (positive vs. negative harms). Counterbalance and participant ID were added as random effects. Again we ran a model predicting number of solutions recommended and number of solutions included in the top 5 most effective.

Number of solutions recommended

There was a main effect of Solution, $F(1, 938.02) = 42.810$, $p < .001$, $partR^2 = .000$, 95% CI [0.006, 0.000]. Participants offered more additive ($EMMean = 3.98$; $SE = .084$) than

subtractive solutions (*EMMean* = 3.70; *SE* = .084). There was also a significant Solution by Participant Gender interaction, $F(1, 938.02) = 8.992$, $p < .001$, $partR^2 = .000$, 95% CI [0.006, 0.000]. However, both Men, $b = 0.149$, *SE* .057, $p = .046$, and Women, $b = 0.402$, $SE = .062$, $p < .001$, suggested significantly more additive than subtractive solutions and they did not differ from one another in how additive or subtractive they were (smallest $p = .662$, for the difference in number of subtractive solutions suggested).

There was also a significant interaction between Solution and Advice Recipient, $F(2, 938.02) = 17.344$, $p < .001$(for the interaction between Solution and Self/Friend/Stranger at the level of friend vs. stranger: $partR^2 = .006$, 95% CI [0.018, 0.000]; for the interaction between Solution and Self/Friend/Stranger at the level of friend vs. self: $partR^2 = .000$, 95% CI [0.005, 0.000]). There was no difference between the number of additive and subtractive solutions recommended to close friends, $b = 0.049$, $SE = .073$, $t(938) = 0.667$, $p = .505$, but people were more additive than subtractive when advising strangers, $b = 0.620$, $SE = .073$, $t(938) = 8.500$, $p < .001$, and themselves, $b = 0.157$, $SE = .073$, $t(938) = 2.155$, $p = .031$. People were significantly more subtractive when advising their friends, $b = 0.691$, $SE = .073$, $t(938) = 9.474$, $p < .001$, and themselves, $b = 0.704$, $SE = .073$, $t(938) = 9.655$, $p <.001$, than they were when advising strangers. People were also significantly more additive when advising themselves than when advising strangers, $b = 0.241$, $SE = .073$, $t(938) = 3.300$, $p = .003$, but there was no difference in the number of additive solutions offered to the self, $b = 0.122$., $SE = .073$, $t(938) = 1.669$, $p = 218$, or strangers, $b = -0.119$, $SE = .073$, $t(938) = -1.633$, $p = .232$, when compared with friends.

People were highly additive to themselves and this differed significantly from the number of subtractive solutions they recommended to themselves and to the number of additive solutions they recommended to strangers.

Ranking of solutions

There was no main effect of Solution, $F(1, 1086) = .030$, $p = .862$, $partR^2 = .000$, 95% CI [0.005, 0.000] (Additive solutions: *EMMean* = 2.34, *SE* = .046; Subtractive solutions: *EMMean* = 2.36, *SE* = .046).

There was, however, a significant interaction between Solution and Advice Recipient, $F(2, 1086) = 12.240$ $p = < .001$, $partR^2 = .010$, 95% CI [0.025, 0.002]. Subtractive solutions were rated within the top 5 most effective solutions significantly more than additive solutions when advising friends, $b = -0.368$, $SE = .113$, $t(903) = -3.262$, $p = .001$. However, when advising strangers, additive solutions were rated within the top5 more than subtractive solutions were, $b = 0.438$, $SE = .113$, $t(903) = 3.876$, $p < .001$. There was no difference between solution types when advising oneself, $b = -0.104$, $SE = .113$, $t(903) = -0.924$, $p = .356$.

There was also a significant interaction between solution type and participant gender, F(1, 1086) = 13.413, $p < .001$, $partR^2 = .007$, 95% CI [0.020, 0.001]. Men included subtractive solutions in their top 5 most effective solutions more than additive solutions, $b = -0.250$, $SE = .0885$, $t(903) = -2.826$, $p = .005$, whereas, women included additive solutions in their top 5 more than subtractive solutions, $b = 0.227$, $SE = .096$, $t(903) = 2.375$, $p = .018$.

Analysis of pre-existing harms

We selected only advice given to strangers and examined the effect of the inclusion of positive vs. negative harms in the vignettes on advice giving.

Number of solutions. There was a main effect of Solution, $F(1, 1298.16) = 233.447$, $p < .001$, $partR^2 = .024$, 95% CI [0.042, 0.011]. Participants offered more additive (*EMMean* = 3.87; *SE* = .089) than subtractive solutions (*EMMean* = 3.25; *SE* = .089).

There was also an interaction between Solution and Participant Gender, $F(1, 1298.16) = 30.889, p < .001, partR^2 = .002$, 95% CI [0.008, 0.001]. However, both men, $b = 0.398$, $SE = .056$, $t(1298) = 7.120, p < .001$, and women, $b = 0.852$, $SE = .060$, $t(1298) = 14.258$, $p < .001$, offered more additive than subtractive solutions.

There was also a significant Solution by Harm interaction, $F(1, 1298.16) = 166.268, p < .001$, $partR^2 = .007$, 95% CI [.019, .001]. Participants suggested more additive than subtractive solutions to people who were engaged in negative harms – activities that harm their mental health through the absence of something beneficial (e.g., not exercising) – $b = 1.1520$, $SE = .058$, $t(1298) = 19.880, p < .001$. However, there was no difference in the number of additive and subtractive solutions suggested to people who were engaged in positive harms – things that are directly detrimental to mental health – $b = 0.098$, $SE = .058$, $t(1298) = 1.690, p = .091$.

There were no significant interactions with Vignette Gender (smallest $p = .168$ for the four-way interaction between Solution, Harm Type, Participant Gender and Vignette Gender).

Ranking of solutions. There was a main effect of Solution, $F(1, 1451) = 56.115, p < .001$, $partR^2 = .036$, 95% CI [0.057, 0.20]. Additive solutions were ranked in the top 5 most effective solutions (*EMMean* = 2.50; *SE* = .043) more than subtractive solutions (*EMMean* = 2.04; *SE* = .043).

There was also an in interaction between Solution and Participant Gender, $F(1, 1451) = 21.439, p < .001, partR^2 = .006$, 95% CI [0.016, 0.001]. However, both men, $b = 0.173$, $SE = .082$, $t(1269) = 2.096$, $p = .036$, and women, $b = 0.732$, $SE = .88$, $t(1269) = 8.293, p < .001$, included more additive than subtractive solutions in their top 5 most effective solutions. There was a Solution by Vignette Gender interaction, $F(1, 1451) = 14.952, p < .001, partR^2 = .000$, 95% CI [0.004, 0.000]. However, again, more additive than subtractive solutions

appeared in the top 5 for vignettes of men, $b = 0.219$, $SE = .0854$, $t(1269) = 2.563$, $p = .011$, and women, $b = 0.686$, $SE = .0854$, $t(1269) = 8.031$, $p <. 001$.

There was an interaction between Solution and Harm, $F(1, 1451) = 91.695$, $p < .001$, $partR^2 = .025$, 95% CI [0.042, 0.011]. There was a significant difference between additive and subtractive solutions for negative harms, $b = 1.030$, $SE = .086$, $t(1269) = 12.035$, $p <.001$, but not for positive harms, $b = -0.126$, $SE = .085$, $t(1269) = -1.478$, $p = .140$.

There was also an interaction between Solution, Harm and Vignette Gender, $F(1, 1451) = 7.760$, $p = .005$, $partR^2 = .002$, 95% CI [0.009, 0.000]. Significantly more additive than subtractive solutions appeared in the top 5 most effective solutions for women engaged in positive harms, $b = 1.095$, $SE = .121$, $t(1269) = 9.038$, $p < .001$, and negative harms, $b = 0.276$, $SE = .120$, $t(1269) = 2.293$, $p = .022$. This was also true for men engaged in negative harms, $b = 0.965$, $SE = 0.121$, $t(1269) = 7.981$, $p < .001$. However, for men engaged in positive harms, subtractive solutions were rated as more effective than additive solutions, $b = -0.527$, $SE = 0.121$, $t(1269) = -4.376$, $p < .001$.

The four way interaction between Solution, Harm Type, Participant Gender and Vignette Gender was not significant, $F(1, 1451) = 0.1839$, $p = 0.668$, $partR^2 = .000$, 95% CI [0.004, 0.000]

Study 8

One model was tested in this examination of whether GPT 4o was similarly additive in the advice that it gave to the vignettes used in earlier studies and whether this differed as a function of vignette gender or the harm type (only positive harms vs. only negative) expressed in the vignettes. Random effects were included for ‘sample’ (with a view to capturing variability between each request to GPT for a response, similar to Participant ID), and for counterbalance.

There was a main effect of Solution, *F*(1, 796.01) = 13139.938, $p < .001$, $partR^2 = .703$, 95% CI [0.722,0.683]. GPT offered more additive (*EMMean* = 12.90; *SE* = .078) than subtractive solutions (*EMMean* = 2.600; *SE* = .078).

There was also an interaction between Solution and Vignette Gender, *F*(1, 796.01) = 4.696, $p = .031$, $partR^2 = .004$, 95% CI [0.013, 0.000]. However, both men, $b = 10.1$, *SE* = .126, *t*(796) = 79.523, $p < .001$, and women, $b = 10.4$, *SE* = .126, *t*(796) = 82.588, $p < .001$, were offered more additive than subtractive solutions.

There was also a significant Solution by Harm interaction, *F*(1, 796.01) = 215.912, $p < .001$, $partR^2 = .050$, 95% CI [0.072, 0.031]. However, GPT suggested more additive than subtractive solutions to people who were engaged in negative harms, $b = 11.56$, *SE* = .126, *t*(796) = 91.446, $p < .001$, and people who were engaged in positive harms, $b = 8.94$, *SE* = .126, *t*(796) = 70.665, $p < .001$.

There were no other significant main or interaction effects with Vignette Gender (smallest $p = .135$ for the three-way interaction between Solution, Harm Type, Vignette Gender).
